# An overview of genetic variants regarding efavirenz‐related dysthymias in HIV‐infected patients: Response to a letter to the editor

**DOI:** 10.1002/clc.23789

**Published:** 2022-02-16

**Authors:** Zahra Hosseini, Roya Tayeb, Saeed Ghodsi, Seyed‐Ali Sadre Bafghi, Reza Mollazadeh

**Affiliations:** ^1^ Department of Research, Tehran Heart Center Tehran University of Medical Sciences Tehran Iran; ^2^ Department of Cardiology, School of Medicine, Imam Khomeini Hospital Complex Tehran University of Medical Sciences Tehran Iran

To the Editor,

We appreciate Dr. Rujittika Mungmunpuntipantip and Dr. Viroj Wiwanitkit for their valuable comment about our article.^1^ We found that chronic exposure to efavirenz (EFV) may be associated with increased premature ventricular contraction (PVC), premature atrial contraction (PAC) as well as decreased heart rate variability (HRV) in young human immunodeficiency virus (HIV) patients.^2^ The impact of underlying genetic risk factors on the development of dysrhythmias has been partially understood. Given this fact, in conjunction with the genetic predisposition of patients who receive EFV, some issues might be addressed. Cytochrome P450 (CYP) 2B6 enzyme metabolizes EFV in the liver principally via hydroxylation pathways and in part through the glucuronidation process. It serves as a regulator of plasma concentrations and pharmacokinetic features of the drug. Thus, several studies have examined the association of these genetic markers with plasma levels of EFV as well as subsequent adverse effects. Since EFV may prolong QT interval through inhibition of delayed rectifier potassium current (IKr), coded via human Ether‐a‐go‐go‐Related Gene, certain CYP2B6 variants may increase this hazard accordingly.^3^

The locus of CYP2B6 is characterized with multiple variations subtending 38 known different alleles, haplotypes and diverse single nucleotide polymorphisms.^4^ In addition to genetic heterogeneity, allelic variations including multiple CYP2B6 polymorphisms may also contribute to altered pharmacokinetics of EFV.^5^ The CYP2B6*1 and CYP2B6*2 alleles convey normal function whereas CYP2B6*6 is assigned as a decreased function haplotype. Furthermore, CYP2B6*18 and CYP2B6*38 deliver no function or are attributed to null phenotype. Nevertheless, a combination of these haplotypes may show conflicting results under EFV‐containing regimens.

Only homozygous carriers of the CYP2B6*6 allele might be at increased risk of EFV‐mediated QT prolongation.^3^ However, data regarding the role of specific genes in vulnerability to other substrates of EFV‐related dysrhythmias such as increased PVC, PAC, and decreased HRV is still lacking. Hence, genetic assays pertaining to the CYP2B6*6 allele are presumably not sufficient to delineate major pharmacogenetic determinants of EFV‐related dysrhythmias. Besides, the clinical significance of such findings remains unclear. In fact, controversy surrounds the diagnostic utility of the CYP2B6*6 allele in guiding the treatment of HIV‐infected patients who receive EFV. Markers of liver injury may serve as potential clues for EFV‐mediated toxicity and in turn to the presence of CYP2B6*6 haplotype.^4^ On the contrary, decreased metabolizing function, liver injury, and other adverse effects including long QT interval might be subclinical. There is also a probable dose–response relationship between EFV and CYP capacity as well as with subsequent dysrhythmias.^3^ However, we analyzed serum levels of liver enzymes including alanine aminotransferase, aspartate aminotransferase, and alkaline phosphatase in both EFV and non‐EFV groups. No significant differences were found between the two groups over the treatment period. Clinical Pharmacogenetics Implementation Consortium guideline for antiretroviral regimens containing EFV recommends a reduction of the dose from 600 to 400 mg per day in patients with homozygous CYP2B6*6.^4^ However, in our study, the median dose of EFV was 400 mg, which declines the importance of genetic evaluation in our study participants.

According to a previous study in a southeast Iranian population, the allelic frequency of CYP2B6*6/*6 (4.8%) was similar to those in Caucasian, Japanese, and Chinese populations.^6^ However, extrapolation of these results to the general population of Iran seems to be controversial. In conclusion, despite the aforementioned pitfalls a separate prospective study should be designed to reveal the potential linkage between CYP2B6 genotype and subsequent dysrhythmias. Besides, a crucial aspect in the design of such an investigation is the dose–response pattern as well as the temporal relationship between exposure and incidence of dysrhythmias.
